# Response to comment on “Endoscopic submucosal dissection for proximal colonic lesions: an effective therapeutic option”

**DOI:** 10.1055/a-2543-1552

**Published:** 2025-04-04

**Authors:** Ludovico Alfarone, Cesare Hassan, Alessandro Repici

**Affiliations:** 1 551905 Endoscopy Unit, IRCCS Humanitas Research Hospital Department of Gastroenterology, Rozzano, Italy; 2 437807 Department of Biomedical Sciences, Humanitas University, Pieve Emanuele, Italy



We appreciate the authors' insightful comments 1 and the opportunity to address their points regarding our study on endoscopic submucosal dissection (ESD) for proximal colonic lesions at high risk of submucosal invasive cancer (SMIC) 2 .

First, if a universal endoscopic mucosal resection (EMR) approach had been adopted for our cohort of 116 proximal colon lesions, 12 patients with low-risk, endoscopically resectable T1 colorectal cancer would have been unnecessarily referred for major surgery. In addition, surgery would still not have been avoided for the seven patients who underwent close follow-up after non-curative ESD, given the R0 resection status and favorable histological risk features, as determined by a multidisciplinary team decision 3 . Consequently, the number needed to treat with ESD to avoid one major surgery in our cohort was approximately six. Notably, there were no recurrences (0/7) among patients managed with close follow-up rather than surgery after non-curative ESD. Overall, a universal EMR strategy would have led to 19 additional surgeries compared with our selective ESD approach. Furthermore, a recent systematic review and meta-analysis of randomized controlled trials reported an overall 5.6% recurrence rate following EMR with margin thermal ablation, and even higher for large lesions at high risk of SMIC 4 . Therefore, a universal EMR strategy would likely have led to a higher rate of additional endoscopic procedures to remove recurrent lesions.

Second, although a universal EMR approach might have resulted in a slightly lower perforation rate, none of the patients in our cohort required surgery for adverse events (AEs), and two-thirds were discharged on the day of the procedure. These results are consistent with recent findings from the largest Western prospective colorectal ESD cohort 5 , in which the surgery rate due to AEs was < 1%, as opposed to the 3.1% cited by the authors based on older studies. These data further underscore the safety of colorectal ESD when performed in expert hands.

Third, supporting the cost-effectiveness of our strategy, Bahin et al. demonstrated that the costs associated with a selective ESD strategy were $4.22 million per 1,000 cases, compared with $4.33 million for a universal EMR strategy for large colorectal non-pedunculated polyps, establishing selective ESD approach as the most cost-effective option 6 .

In conclusion, although we agree that a universal ESD approach for all large proximal colon polyps is unwarranted, the authors’ conclusions underestimate the utility of ESD for proximal colon lesions. The notion that ESD entails significant risks with limited benefits does not reflect the outcomes achieved in expert Western centers today. Thus, considering the results we achieved in terms of efficacy, safety, and hospitalization length, our selective ESD strategy algorithm stands out as a highly cost-effective approach and does not require any modification. Indeed, the choice of resection technique should be tailored to lesion characteristics and to local expertise to minimize risks and optimize results. In skilled hands, ESD remains a safe, effective, and resource-efficient option for selected proximal colon lesions.

**Publication note**

Letters to the editor do not necessarily represent the opinion of the editor or publisher. The editor and publisher reserve the right to not publish letters to the editor, or to publish them abbreviated or in extracts.
